# The multimodal display of rattlesnakes is a deterring signal that works best with sympatric species

**DOI:** 10.1371/journal.pone.0343121

**Published:** 2026-03-11

**Authors:** Océane Da Cunha, Joshua J. Mead, L. Miles Horne

**Affiliations:** Department of Biological Sciences, The University of Texas at El Paso, El Paso, Texas, United States of America; Instituto Butantan, BRAZIL

## Abstract

The rattlesnake rattle is one of the most iconic communication signals in nature, yet its evolutionary function remains poorly understood. To test the long-standing hypothesis that the defensive display of rattlesnakes acts as a deterrent, we developed a 3D-printed robotic rattlesnake capable of displaying the multimodal sensory stimulus produced by a rattlesnake. This robot was presented to 38 species of zoo-housed animals in a series of behavioral trials. Animals displayed aversive response to the signal, suggesting that this multimodal display functions as a deimatic signal by triggering reflexive avoidance response. Sympatric species exhibited even stronger fear response to the display, suggesting an evolved, innate fear to the signal. These results offer insights into how complex antipredator signals can originate and diversify in the animal kingdom.

## Introduction

The rattlesnake rattle is perhaps one of the most emblematic communication signals found in nature. The profound impact of the rattle extends beyond the natural world by leaving a lasting imprint on human culture, where it has been symbolized in various art forms throughout history [1]. Despite the fascination the rattle creates, the evolutionary origins and functions of this signal remain poorly understood [2,3].

The rattle is an extraordinary and uniquely evolved trait that emerged as a singular evolutionary event [4,5], exclusively found in the genera *Crotalus* and *Sistrurus* [6]. Based on phylogenetic analyses, rattlesnakes rapidly diversified as they invaded a variety of habitats [7,8]. Nowadays, rattlesnakes constitute a significant portion of the snake biomass across many habitats in the Western hemisphere, demonstrating their success as a group [9,10]. Given their rapid diversification and ecological success, the rattle has been previously proposed as an important driver of the radiation of rattlesnakes [5]. The rattlesnake rattle is composed of loosely interlocking keratin segments, and its overall structure is conserved across all rattlesnakes species [6]. The rattle’s characteristic sound is produced when rattlesnakes contract highly specialized tailshaker muscles, causing the keratin segments to vibrate rapidly against each other [11]. Because the rattle does not have any obvious morphological precursors [12], scientists have tried to solve the enigma of its evolution by identifying behaviors that may have preceded its development [6,13]. However, even the current function of the rattle remains poorly understood and uncovering the role of the rattle could shed light on its evolutionary origins and adaptative significance.

Two main hypotheses have been proposed regarding the adaptative role of the rattle. The first hypothesis suggests that the rattle evolved as a caudal lure to attract prey [14], a behavior commonly observed in several snake taxa, especially within the Viperidae family [3]. The second hypothesis proposes that the rattle evolved to deter animals potentially dangerous to rattlesnakes, such as large grazing animals [15] or crevice-probing carnivores [16]. The rattle has been proposed to act as a deterrent through two different mechanisms: deimatic signaling [17], which triggers reflexive avoidance, and aposematic signaling, which conveys a warning of potential danger [18]. It is also important to recognize that the rattle is part of multimodal display, i.e., a display conveying information through multiple sensory channels [19]. Indeed, when a rattlesnake rattles, its behavior combines auditory cues (the sound produced by the rattle) with visual signals, such as tail vibration, body posture, and sometimes conspicuous tail coloration. Multimodal displays often enhance communication, especially in high-stakes context, such as predator deterrence [19]. In rattlesnakes, these sensory cues are typically expressed simultaneously and may work together to increase the receiver’s perception of threat. However, direct evidence supporting the hypothesis this multimodal display acts as a deterrent is lacking, especially in controlled settings [2].

The main objective of our study was to test the hypothesis that the multimodal display of rattlesnakes acts as a deterring signal. To do so, we developed a 3D-printed robot rattlesnake and exposed 38 species of zoo-housed animals to three different behavioral trials and scored their behavior. Our aim was not to isolate the acoustic component of the display (rattle sound), but the full defensive display that rattlesnakes produce when threatened. Defensive displays often integrate multimodal signals to enhance communication, and attempts to separate these visual and acoustic components can produce biologically unrealistic stimuli that receivers would not naturally encounter [18–20].

The second objective was to test whether the effectiveness of the multimodal display of rattlesnakes was greater among species sharing their present distribution with rattlesnakes. We expected species sharing their present distribution with rattlesnakes (sympatric) to exhibit a stronger fear response to the defensive display than species that do not share their current distribution with rattlesnakes (allopatric). Because all animals in this study were assumed to be naïve to rattlesnakes due to being in captivity, any observed differences in behavioral responses between sympatric and allopatric species would likely reflect an evolved, potentially innate sensitivity to the defensive signal, rather than learned avoidance. If the signal deters both sympatric and allopatric species, this would support the idea that the rattle functions as a deimatic signal. However, if sympatric species exhibit stronger fear responses than allopatric species, it would suggest that this signal also serves an aposematic function. Together, these possibilities support the hypothesis that the defensive display of rattlesnakes may have a dual function by acting both as a deimatic and aposematic signal, suggesting that the display began as general startle mechanism that gradually evolved as an aposematic warning.

## Materials and methods

### Rattlesnake robot design

**Fig 1:**
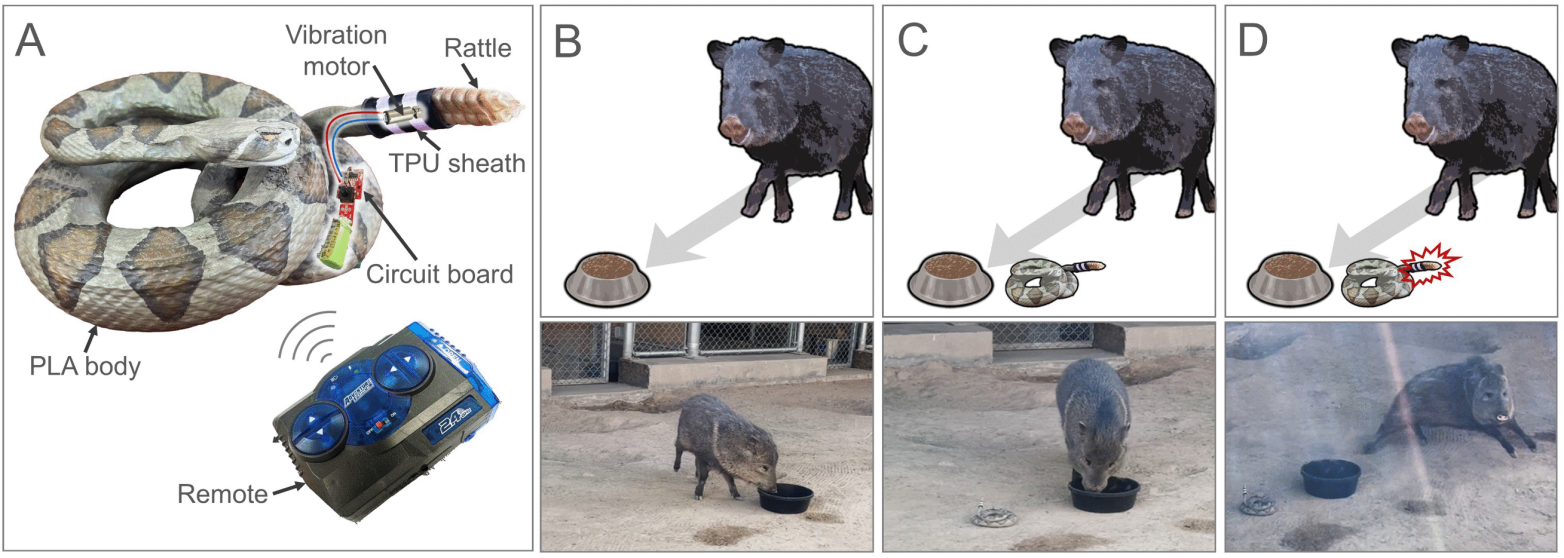
The robotic rattlesnake and experimental set-up. **(A)** Diagram of the 3D-printed robot rattlesnake. **(B-D)** Behavioral responses of a collared peccary (*Pecari tajacu*) during each of the three trial types. In all trials, a food reward was placed in the enclosure to encourage approach. **(B)** The test individual was released in the enclosure after the food reward was placed in the enclosure. **(C)** The robot rattlesnake was placed in the vicinity of the food reward, but the rattle was not triggered when the animal approached the food or the model. **(D)** The robot rattlesnake was placed in the vicinity of the food reward and the rattle was triggered with the remote control when the animal approached the food or the model.

The 3D-printed rattlesnake model was designed and printed by the Fab Lab (501(c)3 Tech and Education Nonprofit Organization) located in El Paso, Texas. A preserved specimen of *Crotalus atrox* from the UTEP Biodiversity Collection (Catalogue #: 12333) was scanned in a defensive position (coiled, head and rattle up). The digital model created from the scan was then adjusted and printed with polylactic acid using a Creality Ender 3 FDM printer (Shenzhen Creality 3D Technology Co., Ltd., Shenzhen, China). The 3D-printed model was approximately 15.5 cm length, 13 cm width, and 7.5 cm height. An interchangeable thermoplastic polyurethane (TPU) sheath was designed to attach at the tail end of the model and hold real rattles. The TPU sheaths were printed in different sizes to accommodate different rattle sizes. Rattles were collected from deceased rattlesnakes (*Crotalus sp.)* found on roads in the El Paso area. To animate the 3D-printed model, the circuit board from a remote-controlled car toy (Model: Stunt Runner; Brand: Adventure force^TM^, Buzz Bee Toys HK Ltd., Mt Laurel, USA) was recovered and refurbished to vibrate the rattle of the 3D-printed rattlesnake model on command. To do so, one of the wheels was replaced by a vibration motor (Tatoko DC 1.5-3V 7,000–14,000 RPM vibrating micro coreless brushed motor). The other wheels were removed from the circuit board. The vibration motor was inserted into the TPU sheath under the rattle and the circuit board was inserted into a compartment within the 3D-printed model, making none of the circuitry visible. The remote had a range of approximately 40 meters and was used to vibrate the rattle during trials. The 3D-printed model was painted according to the coloration and pattern of a western-diamond-backed rattlesnake with acrylic paint (Acrylicos Vallejo, Barcelona, Spain). The TPU sheaths were painted to follow the black and white banding found on the tail of *Crotalus atrox*. The coloration of the robot rattlesnake was designed to provide a general resemblance to *Crotalus atrox* but was not spectrally matched to that species. A layer of varnish (Liquitex professional matte varnish) was applied on the 3D-printed model to protect the paint from damage (Fig 1A).

### Behavioral trials

All behavioral trials adhered to the ethical guidelines of The University of Texas at El Paso and were pre-approved by the University of Texas at El Paso Institutional Animal Care and Use Committee (protocol number: A-201101–1_1701402–1) and by the Association of Zoos and Aquariums (AZA). This study involved only non-human animal subjects; therefore, informed consent from human participants was not applicable.

Behavioral trials were conducted at the El Paso Zoo (TX, USA). To limit stress, trials were conducted in the animal’s home enclosure (indoors or outdoors enclosure). Trials were conducted in the presence of two observers and at least one El Paso Zoo personnel. All animals tested in this study were assumed to be naïve to rattlesnakes as they had spent most or all their lives in captivity. All trials were recorded using a camera (Sony handycam HDR-CX405, Sony, Tokyo, Japan) and the behavioral responses were evaluated from the video records. Three behavioral trials were conducted for each individual. For the first trial (*control*), the test individual was released in the enclosure after a food reward was placed in the enclosure to motivate the animal to come forward. For the second trial (*snake*), the robot rattlesnake was placed about 50 cm from the food reward, but the rattle was not triggered when the animal approached the food or the model. For the third trial (*snake + rattle*), the robot rattlesnake was placed about 50 cm away from the food reward and the rattle was triggered with the remote control when the animal was within one meter of food or the model (Fig 1B-D). The order of the trials was not randomized to minimize the risk of carryover fear response. Trials always started with the *control* trial, then the *snake* trial, and finally the *snake+rattle* trial. The food reward was adapted to the species tested. To minimize olfactory cues and cross-contamination, the robot rattlesnake was sanitized between individuals with a diluted bleach solution. A trial consisting solely of the rattling sound was not included in this study for both theoretical and methodological reasons. First, the primary objective was to evaluate behavioral responses to the combined audiovisual stimulus a rattlesnake would produce, rather than to isolate the contribution of individual sensory modalities (visual and sound). In natural encounters, visual and auditory cues from a rattlesnake often co-occur, especially in close-range interactions, as simulated in this study. We aimed to replicate an ecologically relevant scenario, where rattling is almost always accompanied by the visible presence of a rattlesnake. Second, the acoustic environment of the zoo was challenging as it presented background noises that were highly variable and difficult to control. We were concerned that animals would have difficulty localizing or interpreting a sound in the absence of a visual cue, reducing the likelihood of detecting or responding meaningfully to a sound-only trial. We acknowledge that the same acoustic challenges also apply to the *snake+rattle* trial. However, the rattle was only triggered when animals were within close range of the robot, helping them localize the sound source and associate it with the visual of the robot. For each of these trials, the behavioral response of the individual tested was measured. Individual behavior was scored following a behavioral scale adaptable to any species tested and for all three trials (see Table 1). Specifically, individual behavior was measured using a 4-point ordinal scale reflecting increasing levels of aversion: no reaction, apprehension, startled, and flee. This ordinal structure captured a progression from passiveness (no reaction) to hesitation (apprehension), to defensive or avoidant behavior (startled), to full retreat from the testing area (flee). Two observers who were not present during the trials at the zoo rated the videos independently. Since behavioral scoring required access to both video and audio recordings, raters were not blind to the treatment, potentially introducing observer bias.

**Table 1:** Behavioral scale used to score the behavioral response of animals during trials.

Behavior	Description
No reaction	*Individual readily approached the testing area. Individual did not exhibit any behavior showing that the model was detected if present. Model was detected, individual smells or look at the model but do not show any fear response.*
Apprehension	*Model/food reward approached with caution by the individual. Individual’s body twitched when approaching food reward or model. Individual first ate and then backed up. Individual paused eating and then continued.*
Startled	*Individual either did not approach the model or the food reward or dropped the food reward as soon as the model was detected. Individual was exhibiting threat displays such as vocalizations.*
Flee	*Individual moved away quickly from the model or food reward to completely leave the testing area.*

### Species tested

**Fig 2:**
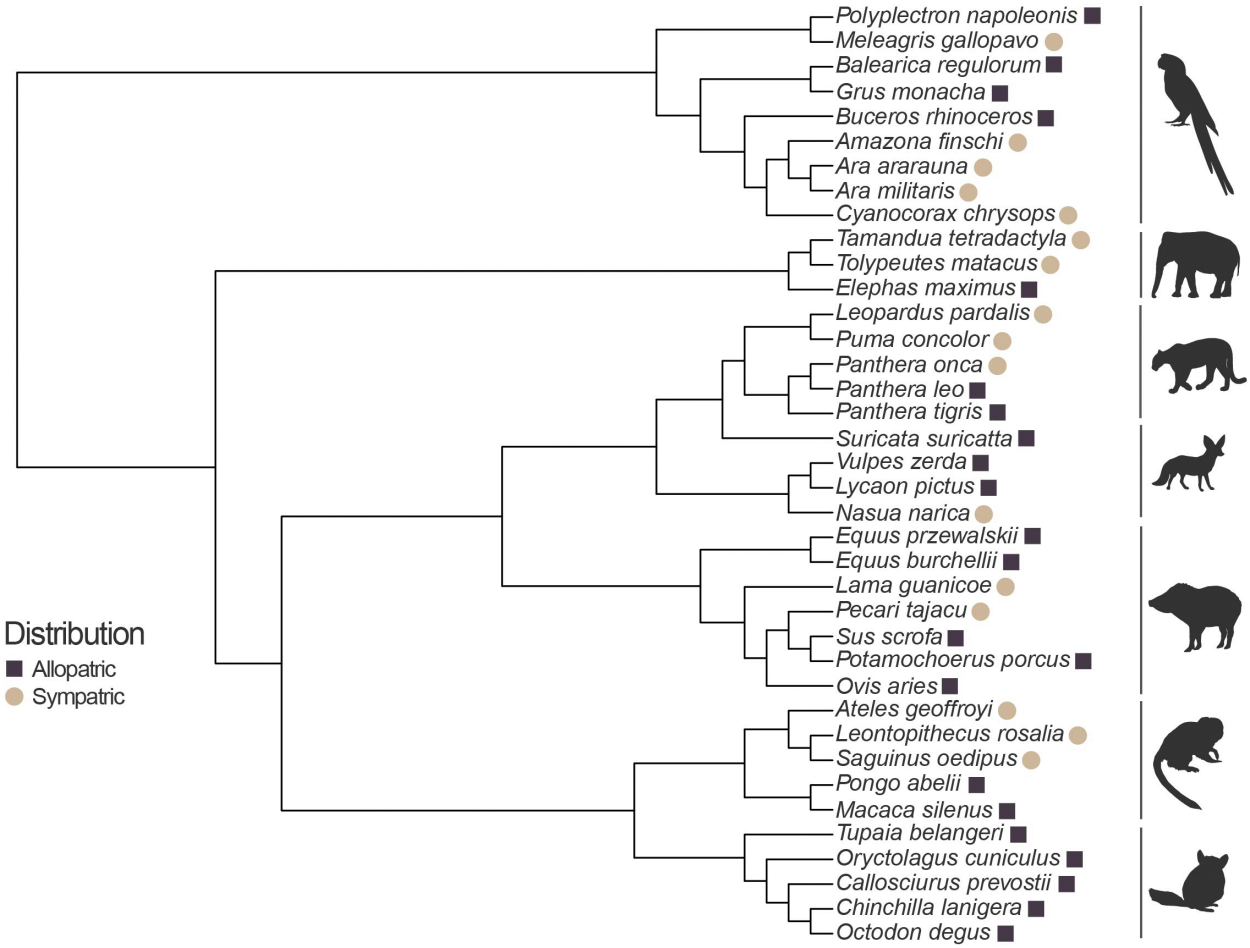
Phylogenetic tree showing the relationship between all species tested for this study. The phylogenetic tree was built using data from the Open Tree of Life. The distribution of species relative to rattlesnake distribution (allopatric/sympatric) is depicted for each species at the end of each branch. Animal silhouettes retrieved from phylopic.org.

The list of species tested is presented in Fig 2. Species were classified as allopatric or sympatric based on their distribution relative to the distribution of extant rattlesnake species. Species distribution ranges were gathered from the IUCN red list [21]. Species were considered sympatric if any part of its range overlapped with at least one species of rattlesnake (*Crotalus sp.* or *Sistrurus sp.*). We tested a total of 38 species, 22 of which were allopatric and 16 of which were sympatric with rattlesnakes. The number of individuals tested per species varied between one individual to seven individuals.

### Statistical analysis

All statistical analysis were performed in R (version 4.2.2) [22]. The inter-rater reliability was estimated using Gwet’s AC1 from the package *irrCAC* [23] for the behavioral scores.

To determine whether evolutionary relationships influence behavioral responses to rattling, the phylogenetic signal among taxa in this study was examined. First, a phylogenetic tree encompassing all taxa tested was created using data from the Open Tree of Life with the package *rotl* [24]*.* The branch lengths for this phylogenetic tree were computed using Grafen’s method [25] using the package *ape* [26]*.* The phylogenetic signal was tested for the behavioral response of each trial type (*control, snake, snake + rattle*) using Pagel’s lambda calculated with the package *phytools* [27]*.*

Cumulative link mixed models were conducted using package *ordinal* [28]*.* Regressions were performed with the behavioral score as response variable and species distribution (sympatric or allopatric) and trial type (*control, snake, snake + rattle*) as explanatory variables. Individual identity was included as a random variable to account for repeated measures of the same individual across treatments. Because of the small sample size, only one explanatory variable at a time was included in each model.

## Results

### Inter-rater reliability and phylogenetic signal

Gwet’s AC1 was estimated to be 0.76357 between the two raters for the behavioral scores. As such, the agreement between the two raters was considered to be substantial [29] and thus, only the behavioral scores from one rater were used for the rest of the analysis. This rater was chosen randomly. The estimated value of Pagel’s λ varied between less than 0.001 for the negative and positive control trials and 0.0245 for the experimental trial, indicating minimal phylogenetic signal in the behavioral response of animals. Therefore, phylogenetic corrections were not included in the following analyses.

### Behavioral responses between trials

**Fig 3:**
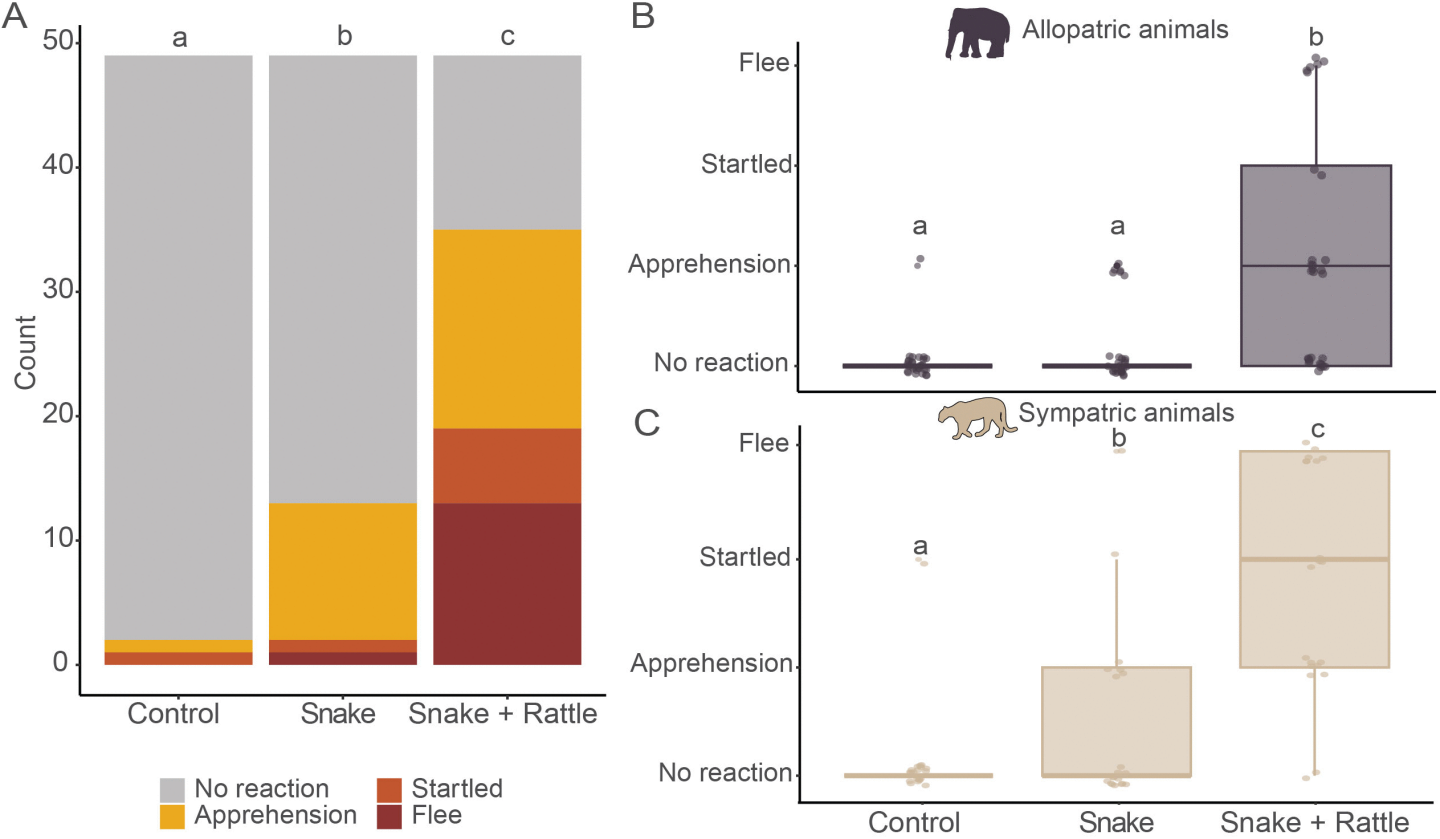
The rattlesnake defensive display acts as a deterrent. Statistical significance between groups is denoted by the letter on top of each bar. **(A)** Behavioral counts per trial type. **(B)** Behavioral responses exhibited by allopatric species for each trial type. **(C)** Behavioral responses exhibited by sympatric species for each trial type.

Cumulative link mixed models indicated that the trial type had a significant effect on the behavioral responses with individuals showing significant stronger fear response in the *snake + rattle* trial (Estimate = 5.50, SE = 0.90, z = 6.10, p < 0.001) and in the *snake* trial (Estimate = 2.68, SE = 0.80, z = 3.35, p < 0.001) compared to the control trial (Fig 3A). Moreover, individuals were significantly more likely to exhibit stronger fear response in the *snake + rattle* than in the *snake* trials (Estimate = 2.81, SE = 0.495, z = 5.68, p < 0.001). For allopatric species, individuals were significantly more likely to exhibit stronger fear response in the *snake + rattle* trial than in the *control* (Estimate = −5.30, SE = 1.2, z = −4.40, p < 0.001) or *snake* trial (Estimate = 3.33, SE = 0.743, z = 4.481, p < 0.001; Fig 3B). For sympatric species, individuals were more significantly more likely to exhibit a stronger fear response in the *snake + rattle* trial (Estimate = −5.95, SE = 1.420, z = −4.20, p < 0.001) and in the *snake* trial (Estimate = −3.34, SE = 0.726, z = 3.601, p < 0.001) than in the control trial (Fig 3C).

### Behavioral responses between sympatric and allopatric species

**Fig 4:**
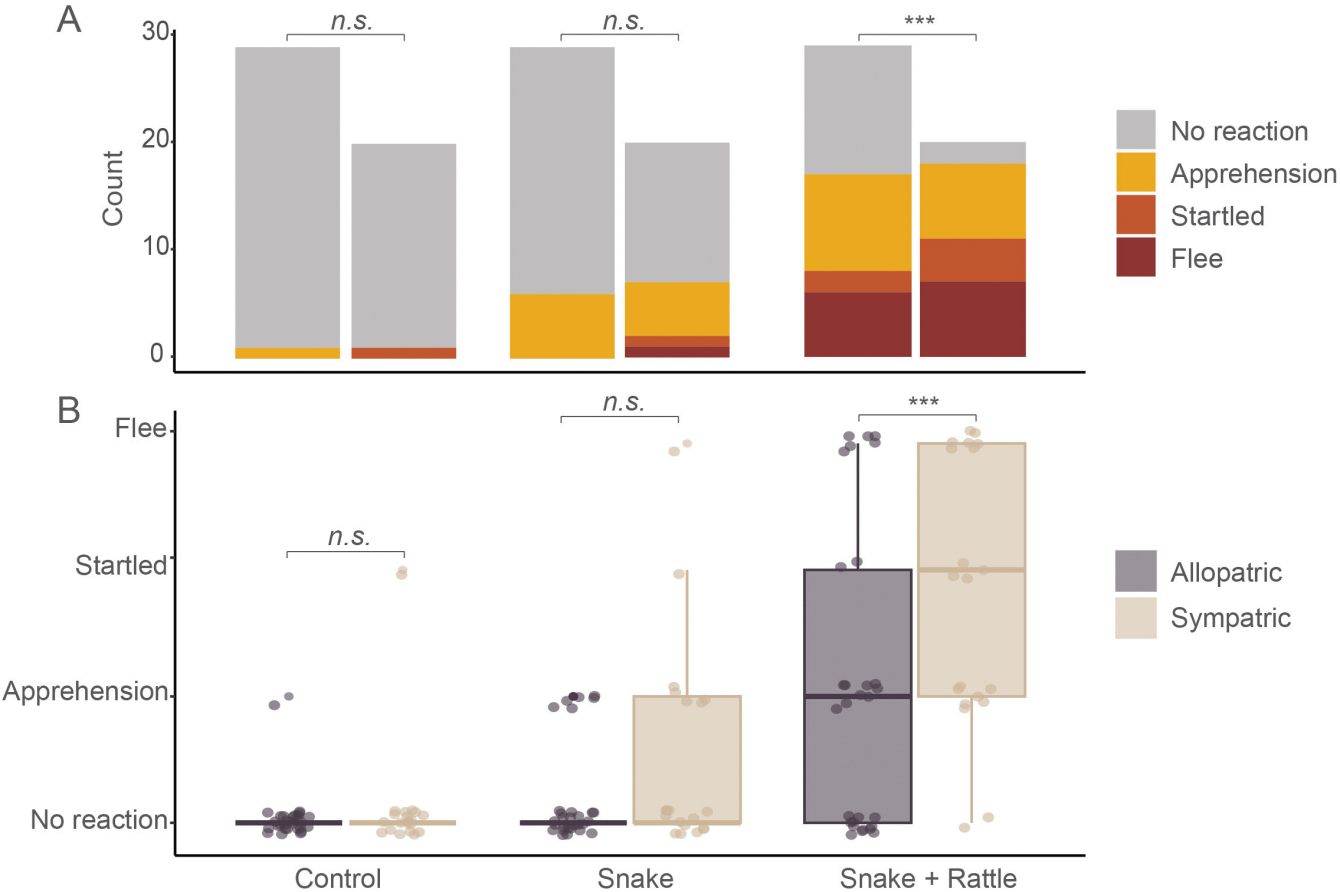
Sympatric species are more sensitive to the rattle than allopatric species. *** denotes statistical significance while *n.s* denotes non-significance. **(A)** Behavior counts per species distribution relative to rattlesnake (allopatric or sympatric) per trial type. **(B)** Behavioral response exhibited by allopatric and sympatric species for each trial type.

Cumulative link mixed models were used to test whether species distribution (allopatric or sympatric) had a significant effect on their behavioral responses for each trial type. Sympatric species were significantly more likely to exhibit stronger fear response than allopatric species during the *snake + rattle* trial (Estimate = 0.59, SE = 0.001, z = 306.7, p < 0.001; Fig 4). On the other hand, no significant differences in behavioral responses were observed between allopatric and sympatric species for the control trial (Estimate = −3.92, SE = 97.15, z = −0.04, p = 0.968) and the *snake* trial (Estimate = 2.60, SE = 1.516, z = 1.716, p = 0.09).

## Discussion

The main objectives of this study were to determine: 1) whether multimodal display of rattlesnakes serves as a deterrent to other animal species and 2) whether its effectiveness is greater among species sharing their present distribution with rattlesnakes. The results of this study demonstrated that a rattlesnake-like multimodal stimulus generally acts as a deterrent, with sympatric species exhibiting stronger fear response than allopatric species.

These results support the long-standing hypothesis that the rattlesnake defensive display is perceived as a deterring signal by other animals and align with anecdotal evidence documented throughout the years [6,30,31] and the limited number of controlled studies that investigated this topic [2,32,33]. These findings are further supported by the fact that rattlesnakes usually rattle in defensive context [34] and that some species mimic rattling behavior as a defensive strategy [35,36]. Indeed, burrowing owls (*Athene cunicularia*) defend themselves against predators by producing hisses that resemble the sound of a rattling rattlesnake [36] and gopher snakes (*Pituophis catenifer*) vibrate their tails similarly to a rattle [35].

While animals exhibited a stronger fear response during the *snake* trial compared to the control trial, their response was even more pronounced during the *snake+rattle* trial. While these results indicate that the full defensive display elicits a stronger fear response, the possibility that animals were responding to a novel sound and object cannot be totally ruled out. Additionally, because the order of the trials was not randomized, accumulated stress across trials could have contributed to the stronger fear response observed in the last trial (*snake + rattle*). Despite this, these results suggest that the rattling signal alters animals’ perception of the robot, leading them to change their behavior. Animals might change their behavior in response to rattling as it triggers a reflexive avoidance response, causing animals to slow or stop their approach, suggesting that the rattle functions as a deimatic signal [18]. The rattle has been previously proposed as a deimatic signal [2,17,18,33] but this hypothesis remained unconfirmed due to a lack of empirical evidence. All animals tested in this study were assumed to be naïve to rattlesnakes as they had spent most or all their lives in captivity. Despite this, the defensive display elicited a wide range of fear responses, from apprehension to fleeing, suggesting that rattling triggers existing reflexive neural mechanisms and works as a deimatic signal. Deimatic behaviors function by triggering reflexive responses such as startle reflexes, looming reflexes, fear responses, sensory overloads, confusions, or neophobia [18,20]. Previous studies have shown that naïve predators respond more strongly to deimatic behavior when compared to experienced predators, potentially because experienced predators learn to ignore or avoid this display [37].

Numerous species of snakes are known to vibrate their tail when threatened [4], an especially common behavior in the families Colubridae and Viperidae, suggesting a shared origin between these families [12]. Given the context in which tail vibration is expressed, tail vibration appears to function as a deterring behavior, likely to be deimatic. Its widespread occurrence and persistence across snake species suggest that it is likely to provide a survival advantage. Ancestral character state reconstructions analyses indicate that the rattlesnake rattle and rattling behavior could have evolved from the widespread tail-vibrating behavior [12]. According to this, the rattle seems to follow the startle-first hypothesis, which states that the act of performing a defensive behavior (in this case, tail vibration) provides protective value and can facilitate the evolution of additional defenses, such as the rattle [20]. This further suggests that rattling functions as a deimatic signal, similar to tail vibration, which may explain why allopatric species and naïve individuals exhibit fear responses when exposed to the rattle. However, this does not explain why sympatric species exhibited significantly stronger reactions to the rattle than allopatric species, even if they were all naïve to rattlesnakes.

Sympatric animals might be more sensitive to the full defensive display than allopatric species because they have evolved an innate fear of the defensive signal, suggesting that this display serves as an aposematic signal for sympatric species. Indeed, while deimatic displays trigger reflexive response that do not require learned or innate aversion, aposematism relies on aversion that is either innate or learned from the focal species itself [20]. The innate avoidance of aposematic species or patterns have been previously found in several species, including humans [38]. For example, hand-reared naïve great kiskadee (*Pitangus sulphuratus*) and turquoise-browed motmots (*Eumomota superciliosa*) both show a strong innate aversion to the aposematic yellow-red pattern of coral snakes [39,40]. While several species of tail-vibrating snakes are non-venomous [12,35,41], all species with a rattle are venomous [6]. What likely began as a deimatic signal through tail vibration may have evolved into an aposematic signal as species started to associate the rattle with the threat of a venomous bite. While avoidance may have initially been a learned behavior, selection may have acted upon individuals with a higher propensity for aversion responses and subsequently evolved into an innate response to the rattle. Evidence showed that aversion can evolve relatively quickly within populations: red-bellied black snakes (*Pseudechis porphyriacus*) developed an innate avoidance for the invasive marine toads (*Rhinella marina*) in Australia in less than 23 generations [42]. While our study does not quantify the strength of rattlesnake impact on selection, this could be a crucial next step in understanding how innate fear become fixed in sympatric populations.

In conclusion, this study demonstrated that the defensive display of rattlesnakes functions as a deterrent signal, a hypothesis widely accepted over time and previously supported, until now, by a few controlled-studies [32,33] and anecdotal evidence [6,30,31]. More importantly, this study highlighted the dual functionality of this display, suggesting that this multimodal signal potentially acts both as a deimatic and aposematic signal. Although the distinction between aposematism and deimatism was not directly tested during this study, the difference in behavioral responses between allopatric and sympatric species allows us to infer potential mechanisms. The defensive display of rattlesnakes effectively deters animals by triggering reflexive avoidance responses regardless of their geographic origin or past experience, supporting the role of the rattle as a deimatic signal. Sympatric species demonstrated significantly stronger fear responses than allopatric species, potentially due to an evolved innate aversion to the rattlesnake display, refining the function of this display as an aposematic signal in these species. This dual signaling strategy provides insight into the evolutionary trajectory of the defensive display of rattlesnakes, suggesting that it likely originated as a general startle mechanism and gradually evolved into a more specialized aposematic warning.

This study raises several intriguing questions, including the role of experience in shaping responses to a deterring signal, the extent of selective pressure required for a deimatic signal to evolve into an aposematic one, and the overall efficiency of this display in preventing predation. Although our study focused on the multimodal stimulus produced by rattlesnakes, future studies should build on these findings to disentangle the contribution of its visual and acoustic components. Such studies would clarify which elements of the display are necessary to elicit deterrence and would provide deeper insight into how multimodal warning signals originate, are maintained, and diversify across lineages.
